# Region Specific Effects of Maternal Immune Activation on Offspring Neuroimmune Function

**DOI:** 10.4236/oji.2015.52006

**Published:** 2015-06-02

**Authors:** Heping Zhou

**Affiliations:** Department of Biological Sciences, Seton Hall University, New Jersey, USA

**Keywords:** Maternal Immune Activation, Lipopolysaccharide, Neuroimmune, Cytokine, Chemokine

## Abstract

Growing evidence suggests that maternal immune activation has a significant impact on the immuno-competence of the offspring. The present study aimed to characterize region-specific effects of maternal immune activation on the offspring’s neuroimmune function. The offspring born to dams treated with saline or lipopolysaccharide (LPS) at gestational day 18 was stimulated with saline or LPS at postnatal day 21, and the mRNA expression of various inflammatory genes in different brain regions of the offspring was analyzed. The offspring born to saline-treated dams exhibited a typical neuroimmune response with elevated levels of cytokines and chemokines following LPS stimulation in all four brain regions examined. In contrast, the offspring born to LPS-treated dams exhibited significantly reduced mRNA induction of cytokines and chemokines following LPS stimulation in the prefrontal cortex but not in the brainstem when compared with pups born to saline-treated dams. Furthermore, the mRNA expression of LPS-induced I-κB*ζ* was significantly attenuated in the prefrontal cortex when compared with pups born to saline-treated dams. These results suggest that maternal LPS may have differential effects on the neuroimmune function in different regions of the offspring brain, and highlight the importance of maternal milieu in the development of neuroimmune function in the offspring.

## 1. Introduction

A well-organized neuroimmune response is critical for the first-line defense against invading microorganisms and restoring homeostasis in the central nervous system (CNS). The pattern recognition receptors such as Toll-like receptors (TLRs) widely expressed in the CNS play an important role in the initiation of a neuroimmune response. Lipopolysaccharide (LPS), a main component of the Gram-negative cell wall, binds to CD14, an LPS co-receptor, and TLR-4, which triggers the activation of MyD88-dependent and independent signaling pathways, leading to the activation of transcription factors such as nuclear factor kappa-light-chain-enhancer of activated B cells (NF-κB), and thereby increasing the expression of cytokines and chemokines [1].

NF-κB consists of a family of transcription factors including p65 and p50. The transcriptional activity of NF-κB is regulated by canonical inhibitors of κB (I-κBs), such as I-κB*α*, which sequester NF-κBs in the cytosol and thereby prevent them from binding to κB target DNA sequences in the promoter region of inflammatory genes such as interleukin (IL)-6 and tumor necrosis factor (TNF)-*α* [2], as well as by non-classical I-κBs such as I-κB*ζ* in the nucleus. Studies have shown that I-κB*ζ* increases the expression of such NF-κB target genes as IL-6 and monocyte chemoattractant protein (MCP)-1 by forming a complex with NF-κB p50 homodimers or facilitating transcription-enhancing nucleosome remodeling in the nucleus of immune cells [3]–[5]. Furthermore, the duration and strength of NF-κB transcriptional activity may also be affected by posttranslational modifications such as ubiquitination, acetylation, methylation, phosphorylation, oxidation/reduction, and prolyl isomerization [6].

There is accumulating evidence that maternal immune activation affects the developing immune and nervous system in the offspring. For example, the offspring born to polyriboinosinic-polyribocytidilic acid (poly I:C)-treated pregnant rats exhibits neural, behavioral, and pharmacological changes relevant to schizophrenia [7]. The cord blood monocytes isolated from neonatal preterm lambs following maternal exposure to LPS exhibit decreased production of IL-6 in response to LPS stimulation when compared with monocytes from preterm control animals [8]. Consistently, the offspring born to LPS-treated pregnant rats exhibits diminished immune response to LPS challenge as compared to the pups born to vehicle-treated rats [9] [10]. These studies suggest that maternal immune stimulation may suppress the offspring’s immune response to infections.

Maternal treatment with LPS has also been found to have region-specific effects on offspring brain. For example, intraperitoneal (i.p.) injection of LPS at 70% gestation significantly increases the level of cell death in the cortex but not in the periventricular white matter of the fetus compared to those injected with vehicles [11]. Maternal immune activation induces region-specific changes in the expression of cytokines in the offspring mouse brain [12]. Previously, we reported that maternal exposure to LPS has a significant impact on offspring neuroinflammation [10]. However, how maternal LPS stimulation affects the offspring’s neuroimmune function in different brain regions is still largely unknown.

This study aimed to examine the regional pattern of the effects that maternal immune activation has on the offspring’s neuroimmune function. Pregnant rats were treated with 500 μg/kg LPS via i.p. injection on gestational day 18 to induce immune activation. The offspring was allowed to develop up to the time of weaning at postnatal day 21 (P 21). The expression of cytokines, chemokines, and other mediators of the TLR-4 signaling pathway in the prefrontal cortex, hippocampus, cerebellum, and brainstem of the offspring at 2 h following stimulation with saline or 250 μg/kg LPS was examined.

## 2. Materials and Methods

### 2.1. Animals

Adult male and female Sprague-Dawley® rats were purchased from Harlan Inc. (Indianapolis, IN), maintained in a temperature- and humidity-controlled facility with a 12-h light/dark cycle, and fed a standard rat diet and water *ad libitum*. Animals were allowed to acclimate to the animal facility for at least 7 days prior to beginning the experiments. Animal studies including animal breeding were conducted with the approval of the Institutional Animal Care and Use Committee (IACUC) at Seton Hall University.

Each male rat (250 – 300 g) was housed with three female rats (200 – 230 g) at night, and the female rats were visually inspected for the presence of a vaginal plug the next morning. The female rat with a vaginal plug was moved to a separate cage under above-mentioned conditions and the day with a vaginal plug found was defined as gestational day 0. On gestational day 18, the pregnant dams were randomly assigned to receive 500 μg/kg LPS (*Salmonella enterica* serovar Typhimurium; Sigma, St. Louis, MO) or saline via intraperitoneal (i.p.) injection. Following injection with LPS or saline, the dams continued to be housed in above-mentioned conditions. After birth, the litter size was culled to 10 wherever applicable, and the offspring was allowed to develop up to the time of weaning at postnatal day (P) 21 when they were randomly assigned to receive one i.p. injection of saline or 250 μg/kg LPS and sacrificed 2 h later. Different brain regions were then dissected and stored at −80 °C for further analyses.

### 2.2. Total RNA Extraction

Total RNA from dissected brain tissues was isolated using the TRIzol reagent (Invitrogen, Grand Island, NY) according to manufacturer’s instructions. The prepared RNA samples were dissolved in RNase-free water and stored at −80 °C.

### 2.3. Semi-Quantitative Reverse Transcriptase-Polymerase Chain Reaction (RT-PCR) Assay

cDNA was synthesized from 2 μg of total RNA using oligo (dT)_12-18_ primer and Moloney Murine Leukemia Virus (M-MLV) reverse transcriptase (Promega, Madison, WI). After cDNA synthesis, PCR amplification was carried out using appropriate sense and antisense primers specific for rat *β*-actin (a house-keeping gene), IL-1*β*, IL-6, Mob-1, KC, CD14, TLR-4, Myd88, NF-κB, I-κB*α*, and I-κB*ζ* synthesized by Eurofins Genomics (Huntville, AL) in a final volume of 20 μl containing 1 μl of cDNA, 1X PCR buffer, 0.2 μM of each sense and anti-sense primer, 0.2 mM of dNTPs, and 0.5 unit of Taq DNA polymerase (Applied Biosystems, Foster City, CA) [10] [13]. The reaction was heated to 94 °C for 5 min, followed by appropriate cycles of denaturation at 94 °C for 30 s, annealing at 57 °C for 30 s, and extension at 72 °C for 30 s. After the final cycle, a 7-min extension step at 72° C was included. PCR products were then run on a 2.0% agarose gel and the gel image was recorded using a UVP GelDoc-It^™^ imaging system (UVP, Upland, CA). The band intensities of genes of interest were digitized using VisionWorks^™^ LS software (UVP, Upland, CA) and normalized against the intensity of *β*-actin in the same sample.

### 2.4. Statistical Analysis

All data were presented as means ± SD. Two-way analysis of variance (ANOVA) was used to analyze the data with maternal LPS treatment and postnatal LPS stimulation as between-subject factors. Bonferroni post-tests were performed if the overall treatment effects were significant. Results with p < 0.05 were considered statistically significant.

## 3. Results

### 3.1. Expression of Cytokines in Different Regions of Offspring Brain Following LPS Stimulation

To evaluate region-specific effects of maternal LPS on the neuroimmune response in the offspring brain, dams were treated with one dose of saline or 500 μg/kg LPS on gestational day 18 via i.p. injection, and the pups were subsequently stimulated with one i.p. injection of saline or 250 μg/kg LPS at P 21. At two hours after the injection, the pups were sacrificed, and prefrontal cortex, cerebellum, hippocampus, and brainstem were dissected. Total RNA was extracted from these tissues and the relative mRNA levels of cytokines were measured using semi-quantitative RT-PCR.

**Figure 1:**
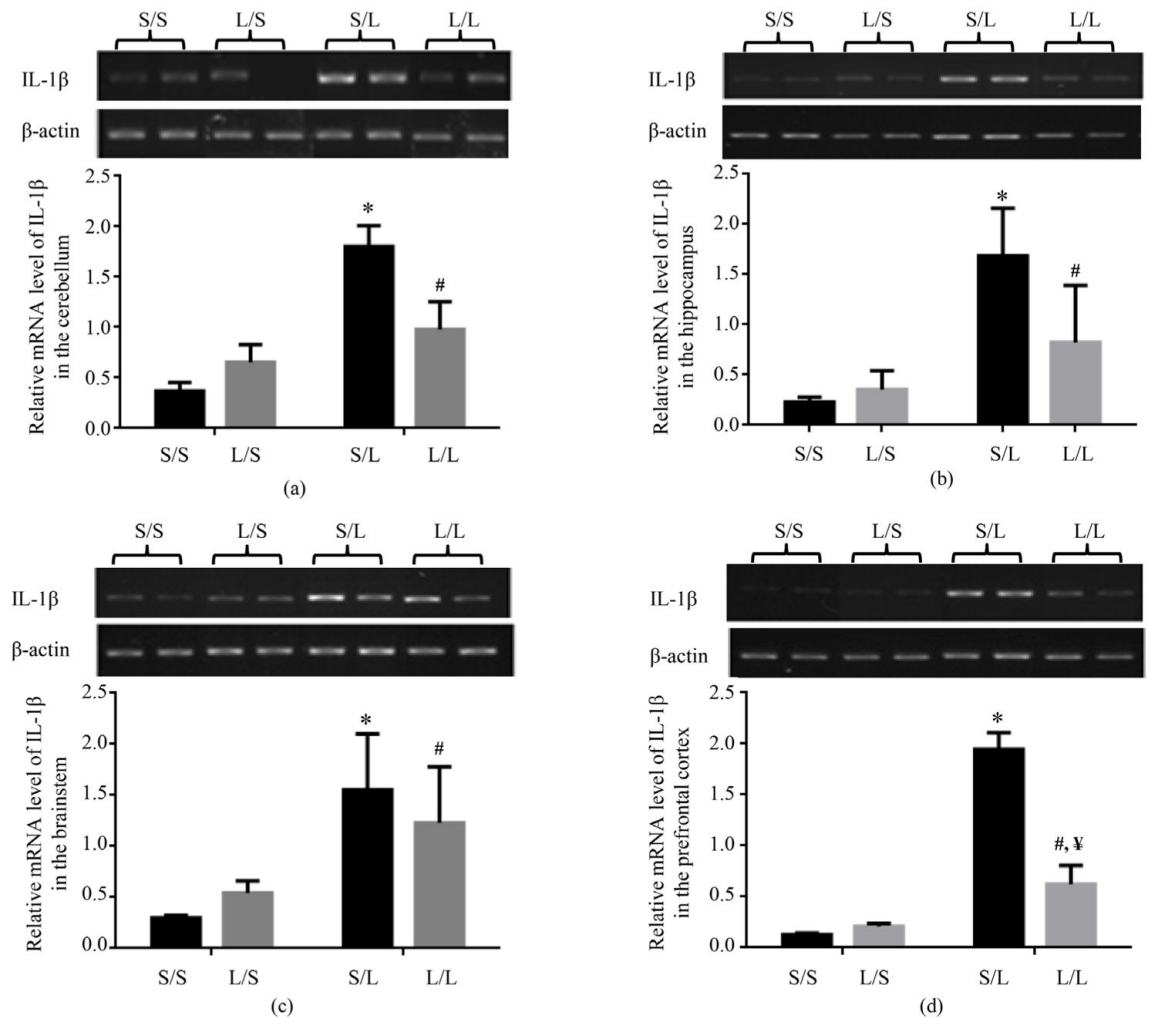
Relative mRNA expression of IL-1*β* in the cerebellum (a), hippocampus (b), brainstem (c), and prefrontal cortex (d) of P 21 pups born to saline or LPS-treated dams at 2 h following stimulation with saline or 250 μg/kg LPS as measured by semi-quantitative RT-PCR. *, vs. S/S; #, vs. L/S; ¥, vs. S/L.

The basal level of mRNA expression of IL-1*β* in the cerebellum (Figure 1(a)), hippocampus (Figure 1(b)), brainstem (Figure 1(c)), and prefrontal cortex (Figure 1(d)) of pups born to dams treated with LPS on gestational day 18 (L/S) trended higher than that in pups born to dams treated with saline (S/S) although the difference was not statistically significant. LPS stimulation significantly elevated the mRNA level of IL-1*β* in the cerebellum, hippocampus, brainstem, and prefrontal cortex of P 21 pups born to dams treated with saline (S/L) and LPS (L/L) as compared to S/S and L/S pups respectively (Figure 1). Furthermore, the mRNA expression of IL-1*β* was significantly lower in the prefrontal cortex of L/L than S/L pups. Although not statistically significant, the mRNA expression of IL-1*β* was dramatically lower in the cerebellum and hippocampus of L/L than S/L pups while IL-1*β* expression in the brainstem of L/L pups appeared to be comparable to that in S/L pups (Figure 1).

**Figure 2:**
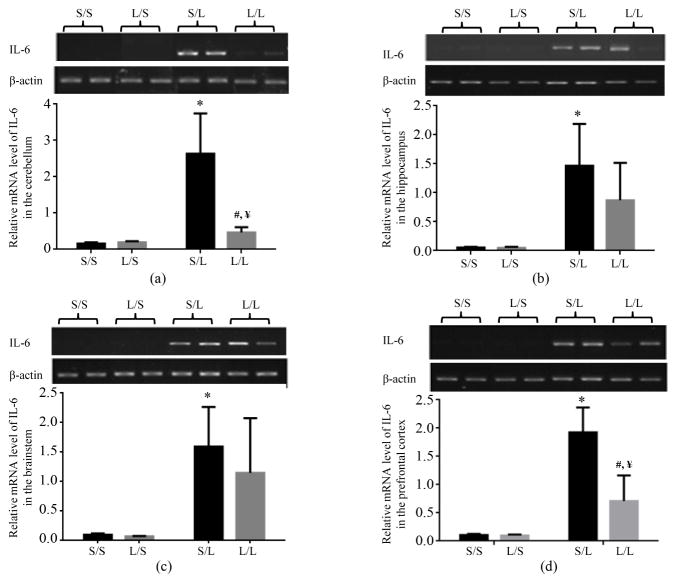
Relative mRNA expression of IL-6 in the cerebellum (a), hippocampus (b), brainstem (c), and prefrontal cortex (d) of P 21 pups born to saline or LPS-treated dams at 2 h following stimulation with saline or 250 μg/kg LPS as measured by semi-quantitative RT-PCR. *, vs. S/S; #, vs. L/S; ¥, vs. S/L.

The mRNA expression of IL-6 in different brain regions of P 21 pups was also examined. The basal mRNA level of IL-6 was very low in all brain regions of S/S and L/S pups. IL-6 mRNA expression was significantly higher in the cerebellum (Figure 2(a)), hippocampus (Figure 2(b)), brainstem (Figure 2(c)), and prefrontal cortex (Figure 2(d)) of S/L than S/S pups. IL-6 mRNA expression was also dramatically higher in all four brain regions of L/L than L/S pups even though the difference in the hippocampus and brainstem did not pass the threshold of statistical significance. Furthermore, the mRNA expression of IL-6 was significantly attenuated in the cerebellum and prefrontal cortex and trended lower in the hippocampus of L/L than S/L pups while it was comparable in the brainstem of S/L and L/L pups (Figure 2).

### 3.2. Expression of Chemokines in Different Regions of Offspring Brain Following LPS Stimulation

**Figure 3:**
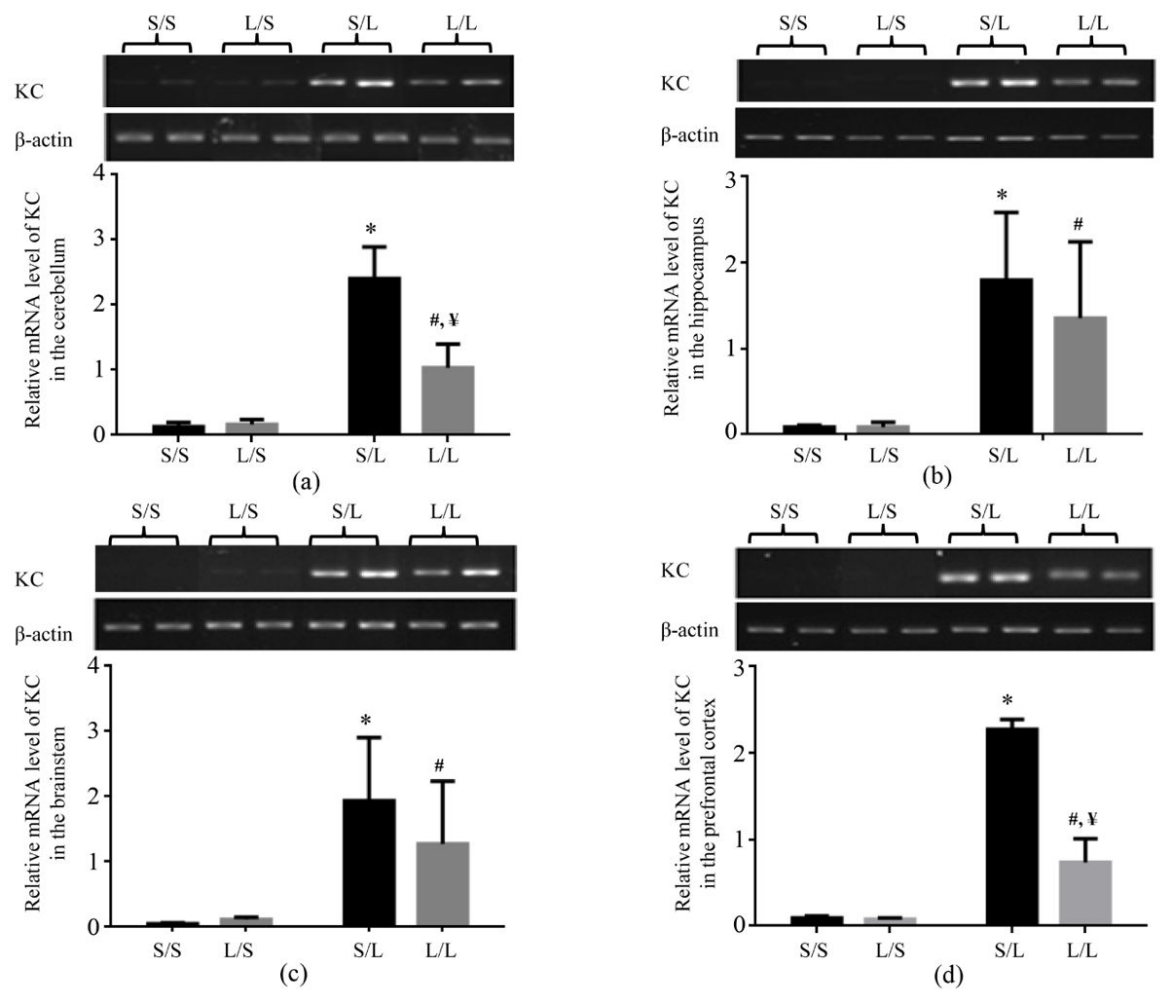
Relative mRNA expression of KC in the cerebellum (a), hippocampus (b), brainstem (c), and prefrontal cortex (d) of P 21 pups born to saline or LPS-treated dams at 2 h following stimulation with saline or 250 μg/kg LPS as measured by semi-quantitative RT-PCR. *, vs. S/S; #, vs. L/S; ¥, vs. S/L.

The expression of chemokines, key soluble factors involved in recruiting immune cells to the brain parenchyma, was then examined. The basal mRNA expression of KC in the cerebellum (Figure 3(a)), hippocampus (Figure 3(b)), brainstem (Figure 3(c)), and prefrontal cortex (Figure 3(d)) of L/S pups appeared to be comparable to that in S/S pups. The mRNA level of KC was significantly higher in the cerebellum, hippocampus, brainstem, and prefrontal cortex of S/L and L/L pups than S/S and L/S pups respectively. Furthermore, the mRNA expression of KC was significantly reduced in the cerebellum and prefrontal cortex, and trended lower in the hippocampus and brainstem of L/L than S/L pups (Figure 3).

**Figure 4:**
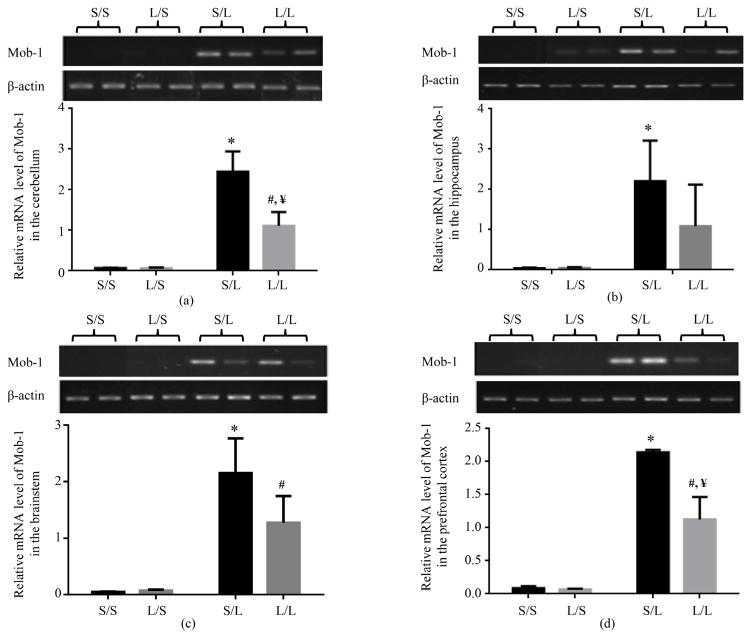
Relative mRNA expression of Mob-1 in the cerebellum (a), hippocampus (b), brainstem (c), and prefrontal cortex (d) of P 21 pups born to saline or LPS-treated dams at 2 h following stimulation with saline or 250 μg/kg LPS as measured by semi-quantitative RT-PCR. *, vs. S/S; #, vs. L/S; ¥, vs. S/L.

The basal level of Mob-1 mRNA expression was very low in the cerebellum (Figure 4(a)), hippocampus (Figure 4(b)), brainstem (Figure 4(c)), and prefrontal cortex (Figure 4(d)) of S/S and L/S pups. LPS stimulation significantly elevated the mRNA expression of Mob-1 in all four brain regions of S/L pups as compared to that in S/S pups. The mRNA expression of Mob-1 was also significantly elevated in the cerebellum, brainstem, and prefrontal cortex, and trended higher in the hippocampus of L/L when compared with L/S pups. Furthermore, the mRNA expression of Mob-1 was significantly attenuated in the cerebellum and prefrontal cortex, and trended lower in the hippocampus and brainstem of L/L when compared with S/L pups (Figure 4).

### 3.3. Expression of Upstream Mediators of TLR-4 Signaling Pathway in Different Regions of Offspring Brain Following LPS Stimulation

**Figure 5:**
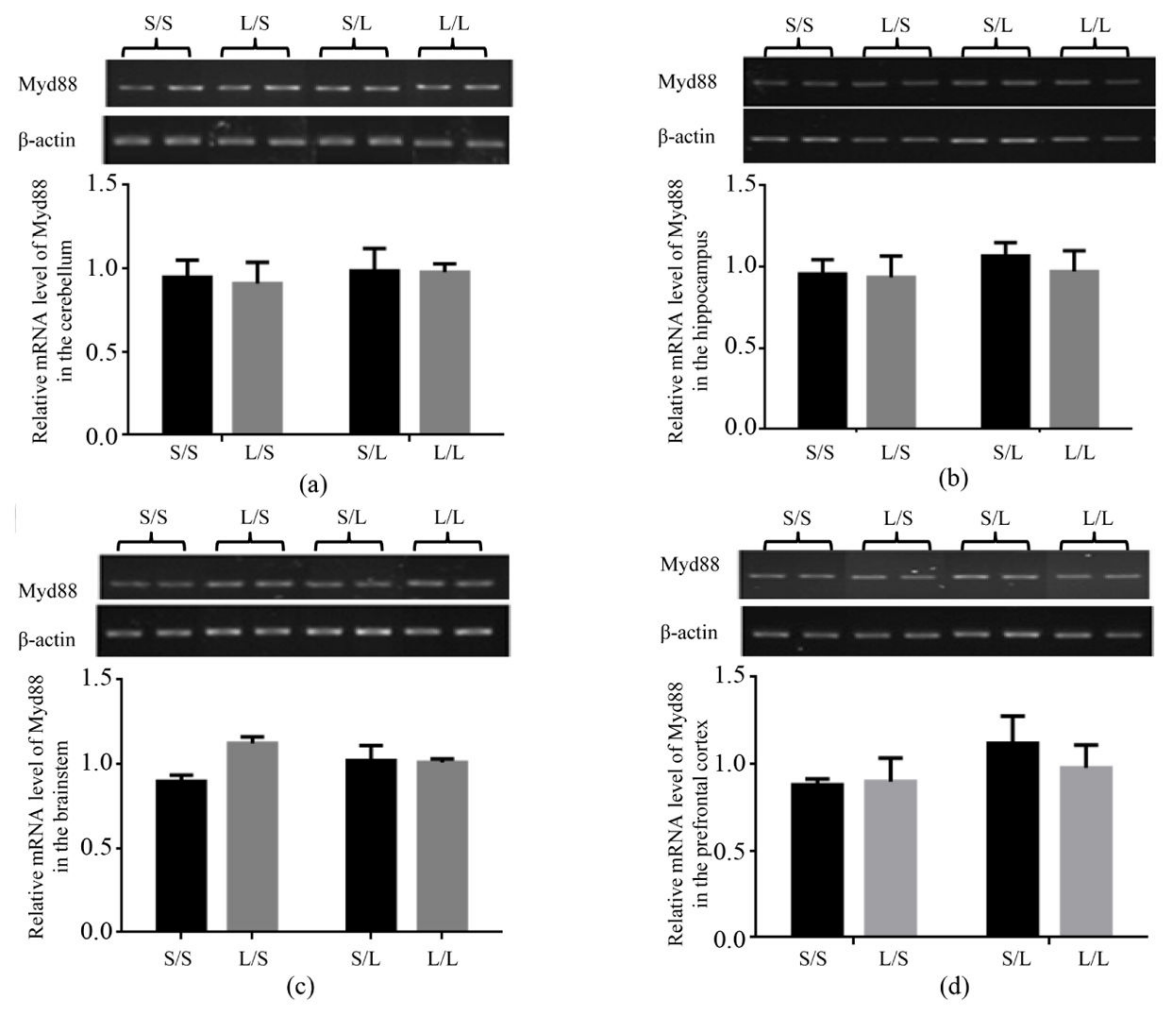
Relative mRNA expression of Myd88 in the cerebellum (a), hippocampus (b), brainstem (c), and prefrontal cortex (d) of P 21 pups born to saline or LPS-treated dams at 2 h following stimulation with saline or 250 μg/kg LPS as measured by semi-quantitative RT-PCR.

**Figure 6:**
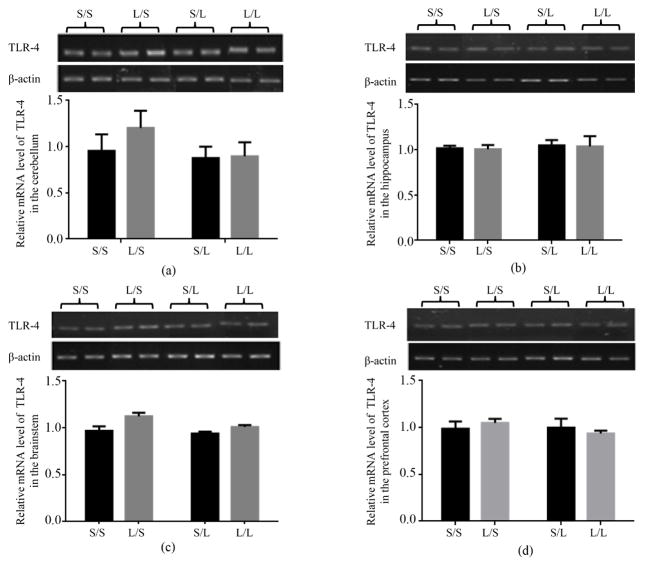
Relative mRNA expression of TLR-4 in the cerebellum (a), hippocampus (b), brainstem (c), and prefrontal cortex (d) of P 21 pups born to saline or LPS-treated dams at 2 h following stimulation with saline or 250 μg/kg LPS as measured by semi-quantitative RT-PCR.

The mRNA expression of upstream mediators of TLR-4 signaling pathway, namely Myd88, CD14 and TLR-4, in different brain regions of the offspring was then examined. Neither maternal nor postnatal LPS significantly changed the mRNA expression of Myd88 in the cerebellum (Figure 5(a)), hippocampus (Figure 5(b)), brainstem (Figure 5(c)), and prefrontal cortex (Figure 5(d)) of the offspring. The mRNA expression of TLR-4 in the cerebellum (Figure 6(a)), hippocampus (Figure 6(b)), brainstem (Figure 6(c)), and prefrontal cortex (Figure 6(d)) was not significantly affected by maternal or postnatal LPS treatment either.

**Figure 7:**
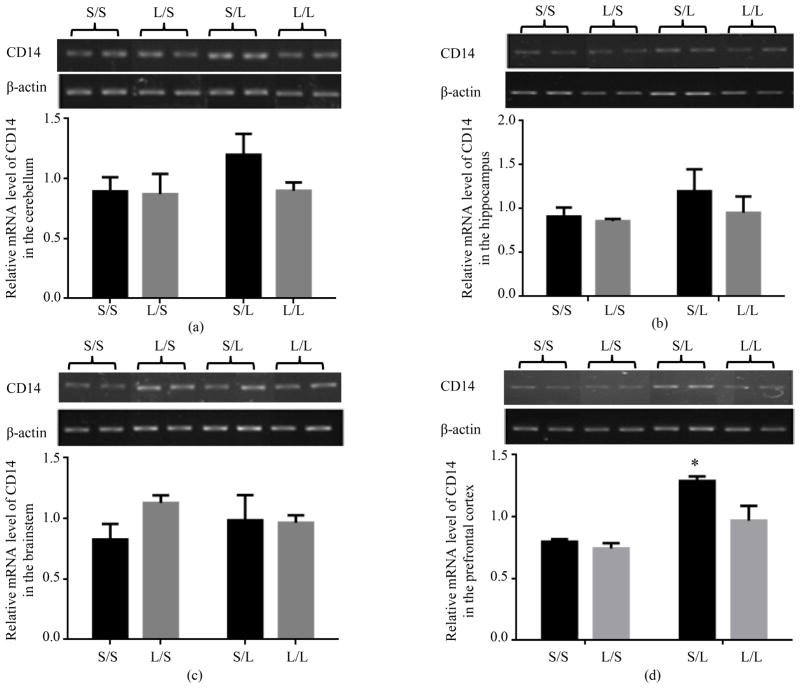
Relative mRNA expression of CD14 in the cerebellum (a), hippocampus (b), brainstem (c), and prefrontal cortex (d) of P 21 pups born to saline or LPS-treated dams at 2 h following stimulation with saline or 250 μg/kg LPS as measured by semi-quantitative RT-PCR. *, vs. S/S.

The mRNA expression of CD14 in the cerebellum (Figure 7(a)), hippocampus (Figure 7(b)), and prefrontal cortex (Figure 7(d)) of S/S pups was not significantly different from that in L/S pups while it trended higher in the brainstem (Figure 7(c)) of L/S than S/S pups. CD14 mRNA expression appeared to be elevated in all four brain regions of S/L when compared with S/S pups, but only the increase in prefrontal cortex was statistically significant. The mRNA expression of CD14 trended higher in the prefrontal cortex of L/L when compared with L/S pups, and was comparable in the cerebellum, hippocampus, and brainstem of L/L pups and L/S pups. Additionally, the mRNA expression of CD14 was lower in the cerebellum, hippocampus, and prefrontal cortex of L/L pups than that in S/L pups, although the difference was not statistically significant (Figure 7).

### 3.4. Expression of Transcription Regulators in Different Regions of Offspring Brain Following LPS Stimulation

**Figure 8:**
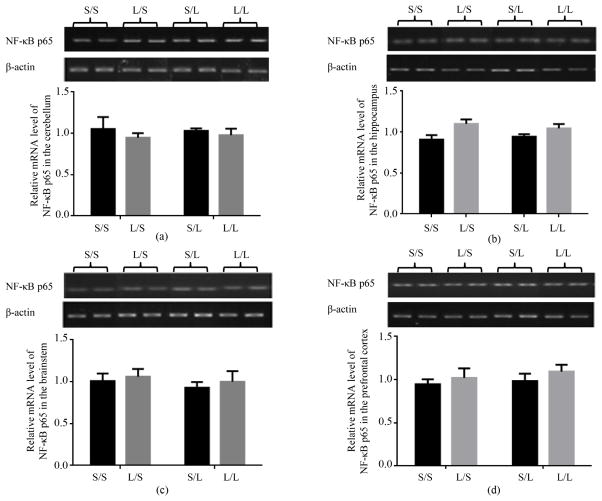
Relative mRNA expression of NF-κB p65 in the cerebellum (a), hippocampus (b), brainstem (c), and prefrontal cortex (d) of P 21 pups born to saline or LPS-treated dams at 2 h following stimulation with saline or 250 μg/kg LPS as measured by semi-quantitative RT-PCR.

**Figure 9:**
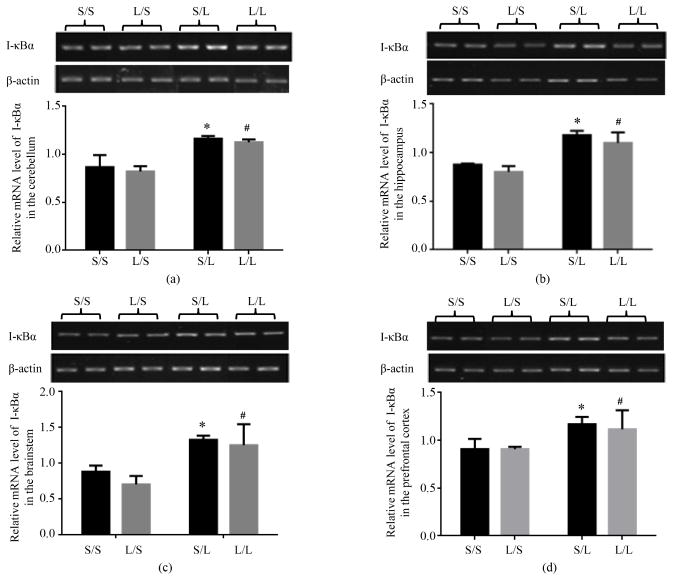
Relative mRNA expression of I-κB*α* in the cerebellum (a), hippocampus (b), brainstem (c), and prefrontal cortex (d) of P 21 pups born to saline or LPS-treated dams at 2 h following stimulation with saline or 250 μg/kg LPS as measured by semi-quantitative RT-PCR. *, vs. S/S; #, vs. L/S.

We then examined the mRNA expression of NF-κB p65 and its regulators in different brain regions of the offspring. The mRNA expression of NF-κB p65 in the cerebellum (Figure 8(a)), hippocampus (Figure 8(b)), brainstem (Figure 8(c)), and prefrontal cortex (Figure 8(d)) of S/S pups was not significantly affected by maternal or postnatal treatment with LPS. The mRNA expression of I-κB*α* in all four brain regions was comparable in S/S and L/S pups, and elevated to equivalent degrees at 2 h following LPS stimulation in S/L and L/L pups (Figure 9).

**Figure 10:**
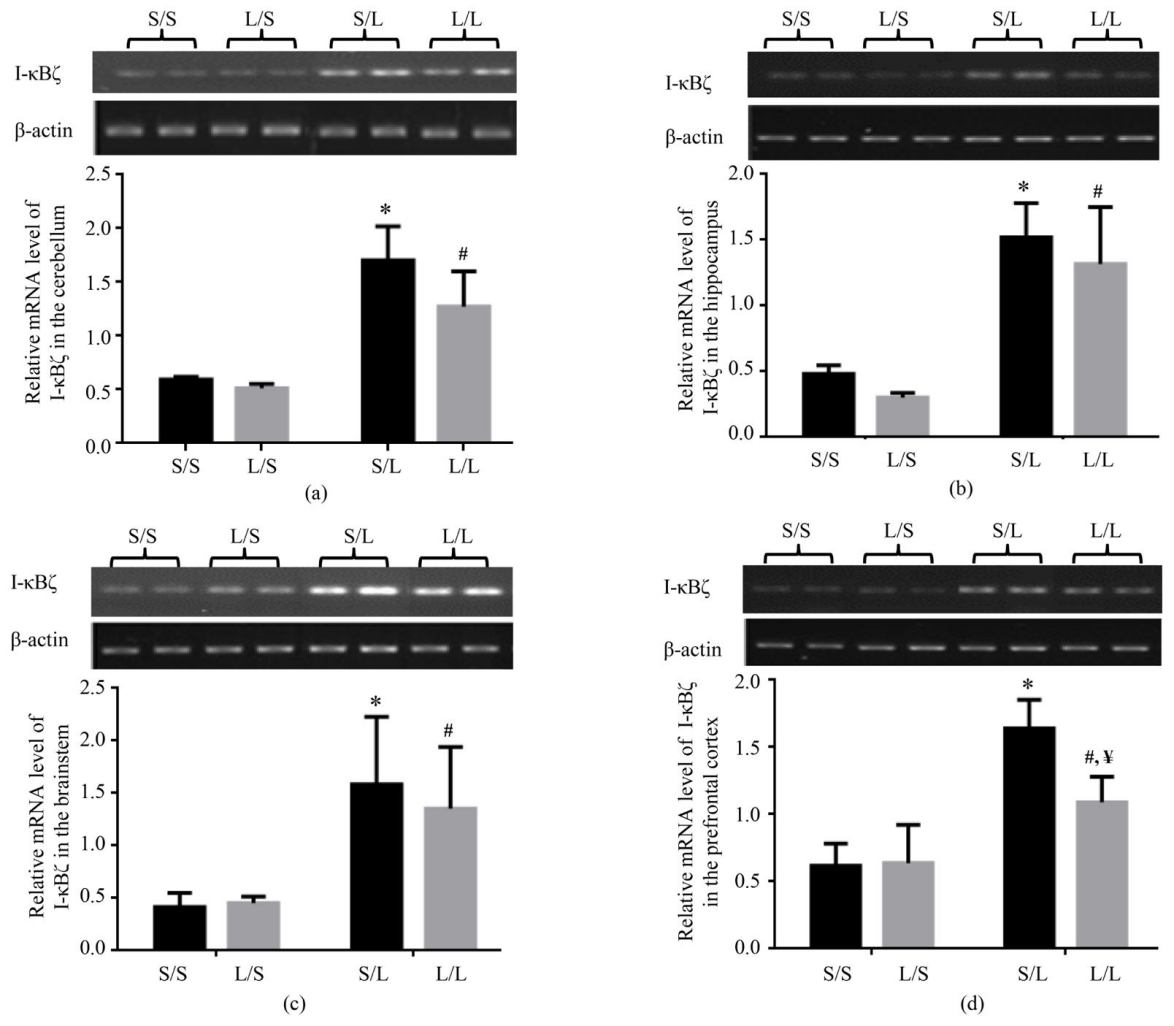
Relative mRNA expression of I-κB*ζ* in the cerebellum (a), hippocampus (b), brainstem (c), and prefrontal cortex (d) of P 21 pups born to saline or LPS-treated dams at 2 h following stimulation with saline or 250 μg/kg LPS as measured by semi-quantitative RT-PCR. *, vs. S/S; #, vs. L/S; ¥, vs. S/L.

The mRNA expression of I-κB*ζ* in the cerebellum (Figure 10(a)), hippocampus (Figure 10(b)), brainstem (Figure 10(c)), and prefrontal cortex (Figure 10(d)) of S/S pups was comparable to that in L/S pups. LPS stimulation significantly elevated the mRNA level of I-κB*ζ* in all four brain regions of S/L and L/L pups. Furthermore, while the mRNA expression of I-κB*ζ* was comparable in the hippocampus and brainstem of S/L and L/L pups, it trended lower in the cerebellum of L/L than S/L pups, and was significantly reduced in the prefrontal cortex of L/L when compared with S/L pups (Figure 10).

## 4. Discussion

Neuroimmune function plays a key role in combating infections, removing debris, promoting repairs, and maintaining homeostasis in the brain. Previous studies have shown that maternal immune activation affects immune as well as neuroimmune responses in the offspring [7] [9] [10]. In this study, we investigated the relationship between maternal immune activation and the neuroimmune function in different regions of the offspring brain. The prefrontal cortex and cerebellum of L/L pups exhibited attenuated mRNA induction of cytokines, namely IL-1*β* and IL-6, and chemokines, namely KC and Mob-1, when compared with S/L pups at 2 h following LPS stimulation even though the difference in IL-1*β* was not statistically significant in the cerebellum. Furthermore, mRNA expression of these cytokines and chemokines in the hippocampus of L/L pups trended lower than that in S/L pups while the mRNA expression of these cytokines and chemokines was not dramatically different in the brainstem of L/L and S/L pups. These findings suggest that the neuroimmune function in four different brain regions of the offspring was susceptible to maternal immune activation to different degrees with prefrontal cortex and cerebellum being the most vulnerable and brainstem the least while the hippocampus was somewhat affected.

In association with reduced mRNA levels of cytokines and chemokines in the prefrontal cortex and cerebellum, the mRNA expression of CD14 in the prefrontal cortex and cerebellum of L/L pups also trended lower than that in S/L pups. Additionally, the mRNA expression of I-κB*ζ* was significantly reduced in the prefrontal cortex and trended lower in the cerebellum of L/L when compared with S/L pups. Studies have shown that I-κB*ζ* is a positive regulator of a subset of NF-κB target genes such as IL-6 [3] [14], which suggests that the reduced induction of I-κB*ζ* in the prefrontal cortex of L/L may have contributed to the attenuated mRNA induction of cy-tokines and chemokines when compared with S/L pups. Further studies on the protein levels of NF-κB and I-κB*ζ* as well as posttranslational modifications of NF-κB would help to better understand how NF-κB regulation contributes to the observed effects of maternal immune activation on offspring neuroimmune function in our animal model.

While the transcriptional activity of NF-κB is regulated by I-κBs, the expression of I -κB*ζ* is, in turn, transcriptionally regulated by NF-κB as part of a feed back loop [15] [16]. Consistently, LPS has been reported to induce the expression of I-κB*ζ* in mouse embryonic NIH-3T3 fibroblast cells [16], human myelomonocytic U937 cells [17], and mouse RAW264 macrophages [18], and in the spleen, lymph node, and lung of mice [14]. Our study also show that LPS stimulation significantly elevated the mRNA expression of I-κB*ζ* in all brain regions of the offspring compared to the saline controls.

A well-organized neuroimmune response is essential for appropriate tissue maintenance and immune surveillance of the CNS, to defend the CNS against pathogens, and to help it recover from stress and injury [19]–[22]. Neuroinflammation has generally been regarded as a double-edged sword that can cause injury to or protect the CNS. There is evidence that neuroinflammation is a risk factor for neurodegenerative disorders, such as Alzheimer’s [23] and Parkinson’s diseases [20] [24]. On the other hand, insufficient neuroinflammation and microglial dysfunction could lead to insufficient clearance of *β*-amyloid plaques and have been proposed as a possible pathway in the pathogenesis of Alzheimer’s disease [25] [26]. Additionally, a neuroimmune-based mechanism has been posited for the etiology of schizophrenia and autism [27]–[30]. Graciarena *et al*. reported that subcutaneous injections of LPS into pregnant rats every other day from gestational days 14 to 20 leads to persistent microglial activation specifically in the hippocampus of adult offspring animals [31]. The present study did examine the mRNA induction of cytokines and chemokines in L/S and S/S pups and found that the mRNA expression of IL-1*β* trended higher in all four brain regions of L/S than S/S pups while the basal level of IL-6, KC, and Mob-1 expression was barely detectable in L/S and S/S pups under the experimental conditions. While it is of interest to further examine the status of neuroinflammation of the offspring at the basal level, the results in this study suggest that the offspring’s neuroimmune response to an immune insult may be impacted to different degrees depending on the brain regions. Considering that neuroinflammation involves finely regulated expression of pro-inflammatory and anti-inflammatory mediators [20]–[22], profiling of neuroinflammatory mediators at different time points following LPS stimulation would help to further delineate the effects of maternal immune activation on the neuroimmune function of the offspring pups and provide a better understanding of the interplay between disturbances in maternal environment and development of neuropathologies in the offspring later in life.

## 5. Conclusion

In summary, this study demonstrated that the prefrontal cortex and cerebellum of L/L pups exhibited attenuated mRNA induction of cytokines, namely IL-1*β* and IL-6, and chemokines, namely KC and Mob-1, when compared with S/L pups at 2 h following LPS stimulation even though the difference in IL-1*β* was not statistically significant in the cerebellum. Furthermore, mRNA expression of these cytokines and chemokines in the hippocampus of L/L pups trended lower than that in S/L pups while the mRNA expression of these cytokines and chemokines was not dramatically different in the brainstem of L/L and S/L pups. In association with reduced mRNA levels of cytokines and chemokines in the prefrontal cortex and cerebellum, the mRNA expression of CD14 in the prefrontal cortex and cerebellum of L/L pups also trended lower than that in S/L pups. Additionally, the mRNA expression of I-κB*ζ* was significantly reduced in the prefrontal cortex and trended lower in the cerebellum of L/L when compared with S/L pups. These findings suggest that the neuroimmune function in four distinct brain regions of the offspring was susceptible to maternal immune activation to different degrees with prefrontal cortex and cerebellum being the most vulnerable and brainstem the least while the hippocampus was somewhat affected, and help to delineate the effects of maternal immune activation on the development of neuropathologies in the offspring later in life.
